# Exploration of a Novel Prognostic Risk Signature and Its Effect on the Immune Response in Nasopharyngeal Carcinoma

**DOI:** 10.3389/fonc.2021.709931

**Published:** 2021-10-07

**Authors:** Shuang Zhao, Xin Dong, Xiaoguang Ni, Lin Li, Xin Lu, Kaitai Zhang, Yanning Gao

**Affiliations:** ^1^ State Key Laboratory of Molecular Oncology, Department of Etiology and Carcinogenesis, National Cancer Center/National Clinical Research Center for Cancer/Cancer Hospital, Chinese Academy of Medical Sciences and Peking Union Medical College, Beijing, China; ^2^ Department of Laboratory, National Cancer Center/National Clinical Research Center for Cancer/Cancer Hospital, Chinese Academy of Medical Sciences and Peking Union Medical College, Beijing, China; ^3^ Department of Endoscopy, National Cancer Center/National Clinical Research Center for Cancer/Cancer Hospital, Chinese Academy of Medical Sciences and Peking Union Medical College, Beijing, China; ^4^ Department of Pathology, National Cancer Center/National Clinical Research Center for Cancer/Cancer Hospital, Chinese Academy of Medical Sciences and Peking Union Medical College, Beijing, China; ^5^ Ludwig Institute for Cancer Research, Nuffield Department of Clinical Medicine, University of Oxford, Oxford, United Kingdom

**Keywords:** nasopharyngeal carcinoma, prognostic signature, biological function, immune response, tumor-infiltrating immune cell

## Abstract

Nasopharyngeal carcinoma (NPC) is a highly invasive and metastatic carcinoma with different molecular characteristics and clinical outcomes. In this work, we aimed to establish a novel gene signature that could predict the prognosis of NPC patients. A total of 13 significant genes between the recurrence/metastasis (RM) group and the no recurrence/metastasis (no-RM) group were identified by machine learning from RNA-Seq data including 60 NPC tumor biopsies. Based on these genes, a 4-mRNA signature (considering U2AF1L5, TMEM265, GLB1L and MLF1) was identified. Receiver operating characteristic (ROC) and Kaplan-Meier (K-M) analyses indicated that this signature had good prognostic value for NPC. The overall survival (OS) and progression-free survival (PFS) of the patients in the high-risk group were significantly shorter than those of the patients in the low-risk group (p = 0.00126 and p = 0.000059, respectively). The area under the ROC curve (AUC) values of the 4-mRNA signature were higher than those of T stage and N stage for OS (0.893 vs 0.619 and 0.582, respectively) and PFS (0.86 vs 0.538 and 0.622, respectively). Furthermore, the 4-mRNA signature was closely associated with cell proliferation and the immune response. The expression of GLB1L and TMEM265 was associated with the level of tumor-infiltrating immune cells (r > 0.4, p < 0.05). We have validated the model through measuring the expression levels of the 4-mRNA signature by qRT-PCR, in an independent cohort of NPC patients. Here, we report a novel gene signature that can serve as a new tool for predicting the prognosis of NPC patients.

## Introduction

Nasopharyngeal carcinoma (NPC) is endemic in Southern China and Southeast Asia (1). Chemoradiotherapy is a highly effective standard treatment for most patients with locoregional disease. However, some NPC patients suffer from distant metastasis and local recurrence after therapy (2). It has been reported that 15% to 60% and 30% to 40% of patients will develop local recurrence and distant metastasis within 4 years after primary treatment, respectively (3–5). Unfortunately, the prognosis of such patients has remained poor because there are no curative options (6, 7). Furthermore, the complex etiologic factors and the high heterogeneity of NPC make prognostic prediction challenging. It is urgent to develop a novel prognostic model for NPC that allows clinicians to employ the appropriate therapeutic strategies for the patients with a favorable prognosis and select the best supportive measures for the patients with an unfavorable prognosis.

The American Joint Committee Cancer (AJCC) tumor-node-metastasis (TNM) staging system is considered to be the standard for prognostic predictions for NPC. However, the value of this system in evaluating the prognosis of NPC patients is limited, because the clinical outcomes are diverse among patients with the same TNM stage who receive similar treatment (8). Thus, molecular markers that can classify patients into groups based on good prognosis and poor prognosis have great clinical value. High expression of EGFR (2, 9), JAK2, and STAT3 and a high copy number of circulating EBV-DNA have been linked with poor survival in patients suffering from NPC (10–12). In addition, noncoding RNAs such as microRNAs and long noncoding RNAs (lncRNAs) have been increasingly reported to be associated with the survival of NPC patients (13, 14). However, the number of prognostic models employing two or more mRNA biomarkers for NPC is limited, but using combinations of markers in such a way could increase specificity and sensitivity. Thus, specific prognostic factors for NPC that predict clinical outcomes and thus improve management are needed.

Tumor progression is a complex process requiring interplay between cancer cells and the microenvironment (15). Some studies have reported that tumor-infiltrating immune cells (TIICs) are associated with clinical outcomes in various cancers (16–19). Additionally, immune checkpoint inhibitors targeting programmed death-ligand 1 (PD-L1) and programmed cell death protein 1 (PD-1) have been developed as new treatment options for patients with cancer (20). NPC is closely associated with Epstein-Barr virus (EBV), and one characteristic pathological finding is massive infiltration of immune cells (21, 22). However, the role of TIICs in the prognosis of NPC is not well known.

As such, we identified a 4-mRNA signature (considering U2AF1L5, TMEM265, GLB1L and MLF1) based on transcriptome data that could estimate prognosis in patients with NPC, and this signature was identified as an independent prognostic factor for NPC. Moreover, we investigated the potential biological relevance of the signature to better understand the relationship between the 4-mRNA signature and prognosis in NPC. Furthermore, we explored the association between TIICs and the prognosis of NPC and the relationship of this 4-mRNA prognostic signature with the host immune response. Overall, this signature could be a prognostic biomarker for NPC and may also reflect immune dysregulation in NPC patients.

## Results

### Significant Genes in the RM and No-RM Groups

In this study, 60 pretreatment, nonmetastatic NPC specimens were included. The clinical characteristics of the patients are listed in **Table 1**. Age, sex, T stage, N stage, and pathological type were not significantly different between the recurrence/metastasis (RM) group and the no recurrence/metastasis (no-RM) group.

**Table 1:** Clinical characteristics of the NPC patients (n = 60).

Variable	no RM group (n = 39)	RM group (n = 21)	p value
Age			0.321^a^
Mean ± S.D.	38 (15.6)	42 (15.6)	
Sex (%)			0.930^b^
Male	26 (66.7)	15 (71.4)	
Female	13 (33.3)	6 (28.6)	
T stage (%)			0.426^c^
1	3 (7.7)	1 (4.8)	
2	8 (20.5)	1 (4.8)	
3	15 (38.5)	10 (47.6)	
4	13 (33.3)	9 (42.9)	
N stage (%)			0.324^c^
0	4 (10.3)	2 (9.5)	
1	8 (20.5)	1 (4.8)	
2	20 (51.3)	11 (52.4)	
3	7 (17.9)	7 (33.3)	
Pathological type* (%)			0.856^b^
undifferentiated	20 (51.3)	9 (45.0)	
differentiated	19 (48.7)	11 (55.0)	

Differences were analyzed by a: student t test, b: Chi-Square test, c: Fisher’s exact test statistical method. *The clinical characteristic has one missing value.

**Figure 1:**
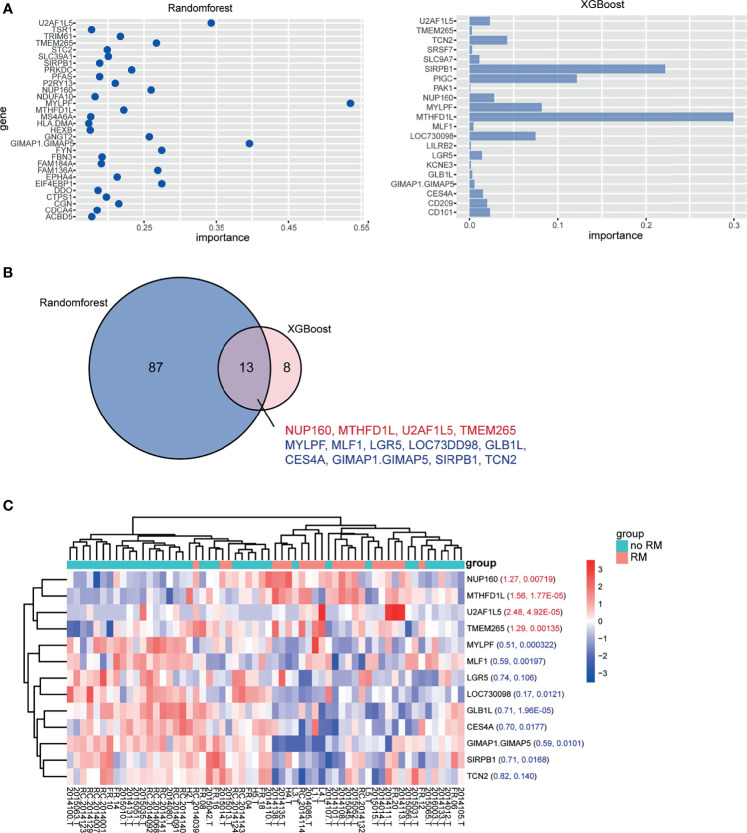
Significant genes in the RM and no-RM groups. **(A)** mRNAs identified in the RM and no-RM groups classified based on the mRNA expression dataset with the RF method (left) and XGBoost method (right). **(B)** Thirteen mRNAs overlapped between the two methods. Red font indicates high expression in the RM group, and blue font indicates low expression in the RM group. **(C)** Heatmap of the expression of the 13 significant genes between the no-RM and RM groups. The foldchange and p value were marked respectively in the heatmap.

We corrected for the effects of age and sex on recurrence and metastasis by constructing a generalized linear model (GLM), and screened 601 genes related to recurrence and metastasis with p < 0.01 (**Table S1**). Two machine learning algorithms, random forest (RF) and extreme gradient boosting (XGBoost), were used. The top 100 genes according to the importance score in the RF results and the most important genes in the XGBoost results (**Figure 1A** and **Tables S2, S3**) were selected, and the intersection was taken. There were 13 genes in total: MYLPF, GIMAP1-GIMAP5, U2AF1L5, TMEM265, NUP160, MTHFD1L, SIRPB1, LGR5, TCN2, GLB1L, MLF1, LOC730098, and CES4A (**Figure 1B**). The expression level of the 13 significant genes between the RM and no-RM groups was visualized (**Figure 1C**).

**Figure 2:**
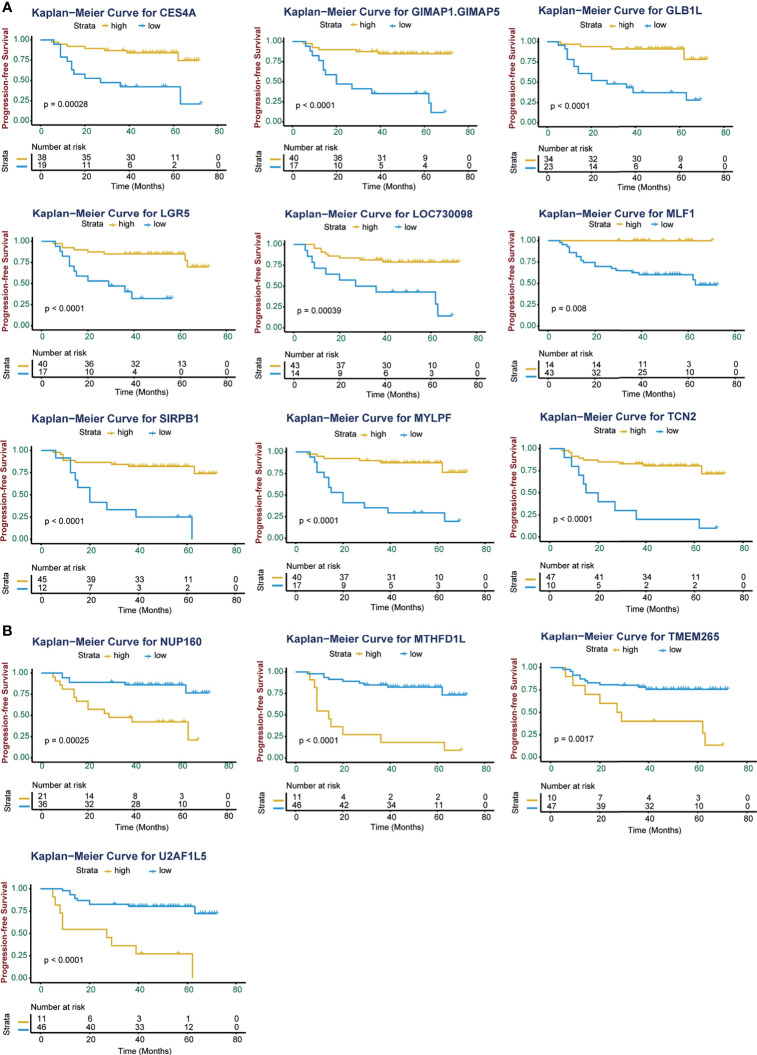
Kaplan-Meier curves of progression-free survival (PFS) for high and low expression of the 13 significant genes in NPC patients. **(A)** High expression level of the genes CES4A, GIMAP1-GIMAP5, GLB1L, LGR5, LOC730098, MLF1, SIRPB1, MYLPF, and TCN2 were associated with improved PFS in patients with NPC. **(B)** High expression levels of the genes NUP160, MTHFD1L, TMEM265, and U2AF1L5 were associated with poor PFS in patients with NPC. The Kaplan-Meier p values are shown.

Kaplan-Meier (K-M) analysis identified high expression of 4 genes (MTHFD1L, NUP160, TMEM265, and U2AF1L5) and low expression of 9 genes (CES4A, GIMAP1-GIMAP5, GLB1L, LGR5, LOC730098, MLF1, MYLPF, SIRPB1, and TCN2) were associated with poor progression-free survival (PFS; p < 0.05; **Figure 2**). Consistent with the PFS results, high expression of the 4 genes and low expression of the 9 genes except LGR5 were linked with poor overall survival (OS; p < 0.05; **Figure S1**). Using univariate Cox regression analysis, 10 of these genes were significantly associated with the prognosis of NPC patients (p < 0.05, **Table 2**). GIMAP1-GIMAP5, MLF1, CES4A, LOC730098, GLB1L, and MYLPF were protective factors (HR < 1), and TMEM265, NUP160, MTHFD1L, and U2AF1L5 were risk factors (HR > 1).

**Table 2:** Univariate analysis of the relationship of gene expression with OS in NPC.

Gene Symbol	p value	HR	Low 95%CI	High 95%CI
TMEM265	0.021	6.42	1.33	30.95
NUP160	0.049	5.75	1.00	32.92
MTHFD1L	0.029	2.95	1.12	7.81
U2AF1L5	0.011	2.26	1.21	4.22
GIMAP1.GIMAP5	0.033	0.64	0.42	0.96
MLF1	<0.001	0.41	0.25	0.68
CES4A	0.007	0.36	0.17	0.76
LOC730098	0.031	0.24	0.07	0.88
GLB1L	0.007	0.18	0.05	0.63
MYLPF	0.020	0.02	0.0008	0.55

HR, hazard ratio; 95%CI, 95% confidence interval.

### Construction of a Prognostic Signature

**Figure 3:**
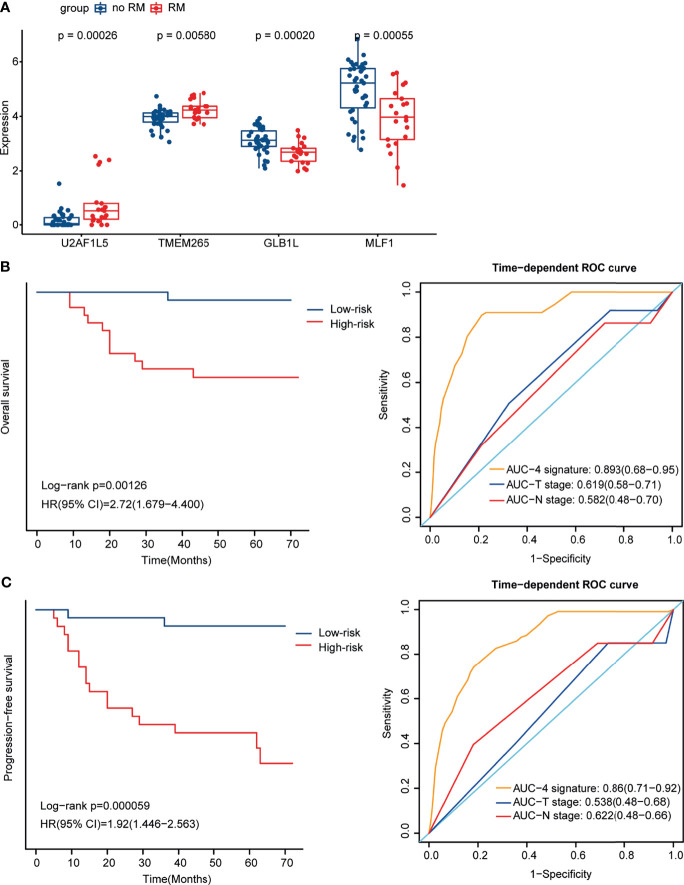
Identification of 4 mRNAs by Multivariate Cox regression analysis and construction of the prognostic signature for NPC. **(A)** Box plot showing the expression level of the 4 mRNAs in the RM and no-RM groups. **(B)** Kaplan-Meier curves for OS for the high- and low-risk groups (left) and ROC curves for the 4-mRNA signature, T stage and N stage for OS (right). **(C)** Kaplan-Meier curves for PFS for the high- and low-risk groups (left) and ROC curves for the 4-mRNA signature, T stage and N stage for PFS (right).

To better estimate the prognosis of NPC, we constructed a prognostic model. After multivariate Cox regression analysis, 4 genes, U2AF1L5, TMEM265, GLB1L and MLF1, were identified and used to construct a prognostic signature for NPC. The expression level of the 4 mRNAs was significantly different between the RM and no-RM groups (**Figure 3A**). We also found that the expression levels of GLB1L and MLF1 were downregulated in tumor tissues relative to normal tissues, which suggested that both of them influence tumor initiation and progression in NPC (**Figure S2**). Based on the median risk scores, 60 NPC patients were classified into a high-risk group and a low-risk group.

To examine the ability of the 4-mRNA signature to estimate the prognosis of NPC, K-M analysis was utilized to evaluate PFS and OS in the high- and low-risk groups. The OS and PFS of the patients in the high-risk group were significantly poorer than those of the patients in the low-risk group (p = 0.00126 and p = 0.000059, respectively); and the area under the receiver operating characteristic (ROC) curve (AUC) values of the 4-mRNA signature were higher than those of T stage and N stage for OS (0.893 vs 0.619 and 0.582, respectively) and PFS (0.86 vs 0.538 and 0.622, respectively). The K-M curves and ROC curves for OS and PFS are shown (**Figures 3B, C** **)**. With an increase in risk score, the number of NPC patients who had died and the number of NPC patients in the RM group increased, which indicated that the outcome of patients with a high risk score was poorer (**Figures S3A, B**). The expression pattern of the 4-mRNA signature in the 60 NPC patients is shown in **Figure S3C**. Of the four mRNAs, two were protective mRNAs (GLB1L and MLF1) whose high expression was associated with better prognosis. In contrast, high expression of the remaining two mRNAs (U2AF1L5 and TMEM265) was associated with poor outcomes.

The results of the multivariate Cox regression analysis showed that the risk score might be an independent predictor of OS and PFS after adjusting for age, sex, T stage and N stage (**Figures S3D, E**). In addition, we used qRT-PCR to identify the expression levels of 4-gene signature (U2AF1L5, GLB1L, MLF1, TMEM265) in an independent cohort (n = 40), which divided into RM (n = 19) and no-RM group (n = 21). The trend of U2AF1L5, GLB1L, TMEM264, MLF1 and risk score is consistent with the NPC RNA-seq cohort (**Figure S4**). The p value in U2AF1L5, MLF1 and risk score is 0.022, 0.042 and 0.039 respectively, but the others is not significant due to the number of cases is small.

### Biological Relevance of the Signature in NPC

**Figure 4:**
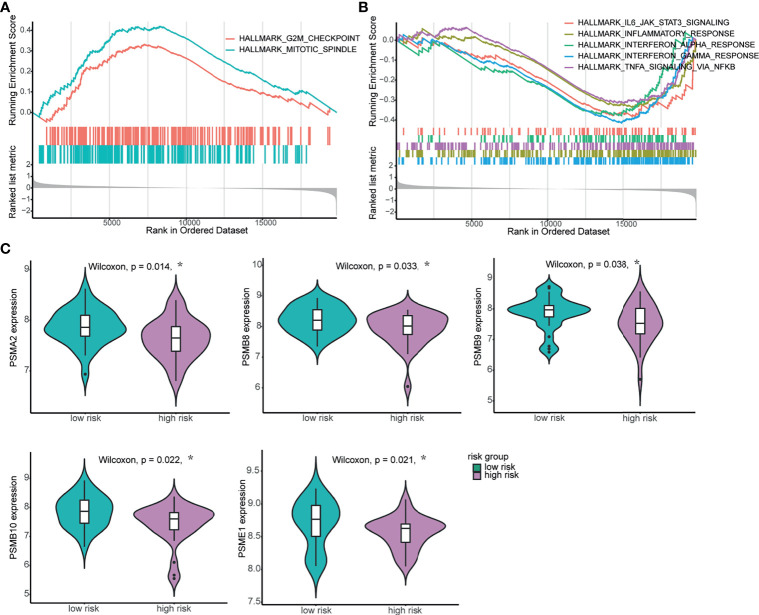
Biological relevance of the 4-mRNA signature in NPC. **(A, B)** GSEA of biological pathways correlated with the 4-mRNA signature. **(A)** The proliferation-related hallmark gene sets “mitotic spindle” and “G2/M checkpoint” were enriched. **(B)** The immune response-related hallmark gene sets “IL-6-JAK-STATS signaling”, “inflammatory response”, “interferon alpha response”, “interferon gamma response” and “TNF signaling *via* NF-ĸB” were enriched. **(C)** The expression levels of genes related to the IFN-γ response between the high-risk and low-risk groups. *p < 0.05.

To further investigate the potential biological underpinnings of the 4-mRNA signature for prognosis, we performed gene set enrichment analysis (GSEA) of the transcriptome data of the 60 NPC tumor samples. As shown in **Figure S5A**, compared to the low-risk group, the high-risk group showed activation of the terms “mitotic spindle” and “G2/M checkpoint” and inhibition of the terms “oxidative phosphorylation”, “interferon gamma response”, “interferon alpha response”, “TNFA signaling *via* NF-κB”, “inflammatory response”, etc. The gene sets with the highest enrichment scores were all closely associated with proliferation and the host immune response (**Figures 4A, B** and **Table S4**). Furthermore, we compared the expression level of genes related to the “interferon gamma response” and found that the expression of most genes, such as PSMA2, PSMB8, PSMB9, PSMB10 and PSME1, was higher in the low-risk group (**Figure 4C**). We also compared the expression of cell proliferation- and cell cycle-related genes between the two groups, and the expression of these genes was higher in the high-risk group (**Figure S5B**). In summary, these results imply that the 4-mRNA signature is related to proliferation and the immune response.

### The Effect of TIICs on the Prognosis of NPC

**Figure 5:**
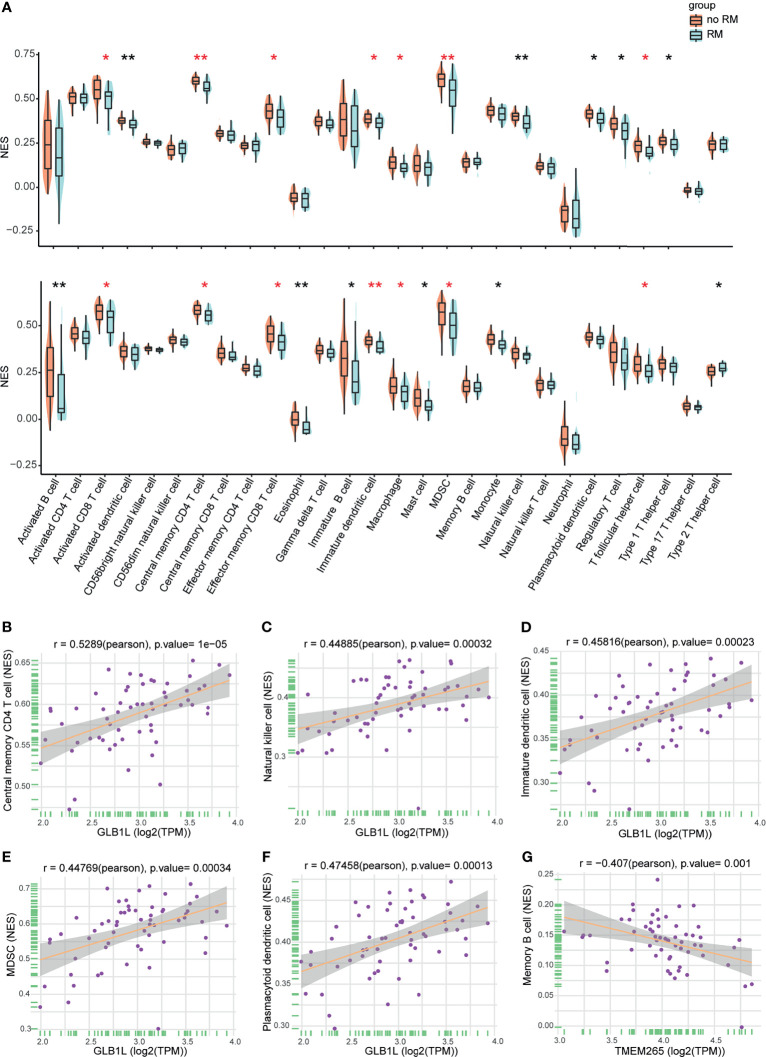
Associations of infiltrating immune cells with prognosis and the signature in NPC. **(A)** NESs of infiltrating immune cells in the RM and no-RM groups (top). NESs of infiltrating immune cells in the RM and no-RM groups of the GEO cohort (bottom). Red indicates statistical differences in both datasets. *p < 0.05, **p < 0.01. **(B–F)** The expression level of GLB1L was positively correlated with infiltrating immune cells. **(G)** The expression level of TMEM265 was negatively correlated with infiltrating immune cells. A correlation coefficient > 0.4 was used.

The tumor microenvironment includes tumor cells, stromal cells, and infiltrating immune cells. In the enrichment analysis, immune response-related hallmark gene sets such as “interferon gamma response” and “inflammatory response” were inhibited in the high-risk group. This result may indicate that the host immune response plays an important role in NPC and that the immune status can estimate the prognosis of NPC. Therefore, we determined whether TIICs are related to the prognosis of NPC. We first compared the tumor purity between the no-RM and RM groups. Higher tumor purity and lower immune score were found in the no-RM group, indicating they were associated with NPC prognosis (**Figure S6**). Then, we utilized single-sample GSEA (ssGSEA) to calculate the normalized enrichment scores (NESs) of 28 infiltrating immune cells in 60 NPC samples, and found more immune cells in the no-RM group than in the RM group. We also performed this analysis on the GEO dataset GSE102349, and the same results were found (**Figure 5A**). These results suggest that TIICs are associated with the prognosis of patients with NPC, especially activated CD8^+^ T cells, central memory CD4^+^ T cells, effector memory CD8^+^ T cells, immature dendritic cells, macrophages, myeloid-derived suppressor cells (MDSCs) and T follicular helper cells, which were statistically different in both datasets. Of note, the role of MDSCs in immune regulation is controversial. Some investigations have indicated that MDSCs can inhibit immune function and are associated with worse prognosis in certain types of cancer (23–25). However, we found that they were associated with favorable prognosis in NPC, which suggests that these cells have a potential role in inducing antitumor immune responses in NPC.

### Correlations of the Signature With TIICs

Based on the above results, we wanted to further explore the role of the 4 mRNAs in the signature in the immune response. The results of the correlation analysis indicated that the expression level of GLB1L was positively correlated with TIICs, including plasmacytoid dendritic cells, immature dendritic cells, MDSCs, activated B cells, natural killer (NK) cells and central memory CD4^+^ T cells **(** **Figures 5B–F** **),** and the expression level of TMEM265 was negatively correlated with TIICs, such as memory B cells (**Figure 5G**). These results may indicate that these two genes affect the prognosis of NPC patients by regulating the host immune response. Furthermore, we found that infiltrating immune cells increased as the expression level of GLB1L increased (**Figure S7A**). Additionally, the association of GLB1L expression with immune cell markers was analyzed with transcriptome data. The results indicated that GLB1L was related to immune markers (**Figure S7B**).

## Discussion

The prognosis of patients with recurrent/metastatic NPC treated with standard chemoradiotherapy is poor. Although numerous studies using microarrays, RNA sequencing and genomic sequencing have been conducted to discover novel biomarkers for predicting the prognosis of NPC (26–28), the management of recurrent/metastatic NPC is a major challenge in the clinic. In this investment, we constructed a 4-mRNA signature considering U2AF1L5, TMEM265, GLB1L and MLF1. The signature could divide NPC patients into high-risk and low-risk groups with different prognoses. Tumor risk stratification tools are significantly important for personalized treatment. Active surveillance is an appropriate choice for patients in low-risk groups, while patients in high-risk groups may require adjuvant therapies. Thus, our 4-mRNA signature may aid the selection of an optimal management strategy and thus avoid unnecessary overtreatment.

In the current study, we also conducted GSEA to further explore the biological relevance of the 4-mRNA signature. Cell proliferation-related hallmarks, such as the terms “mitotic spindle”, “G2/M checkpoint”, and “DNA repair”, and immune-related hallmarks, such as the terms “interferon gamma response” and “inflammatory response”, were enriched. In previous literature, the studied biomarkers have been shown with prognostic impact in various malignancies (29–31). Among them, the role of GLB1L and TMEM265 is consistent with our study, but the mechanism is unknown. MLF1 encodes an oncoprotein which is thought to play a role in the phenotypic determination of hemopoetic cells. However, Rangrez et al. reported that the overexpression of MLF1 inhibited cell proliferation and promoted apoptosis by upregulated the expression of D cyclins, which is consistent with our study (32). What’ more, Chakravorty et al. classified EBV-positive tumor types into two groups (IFN^+^ and IFN^-^), and NPC cases fell within the IFN^+^ group and were characterized by an activated IFN signature. This finding may indicate that the IFN response plays a role in the process of NPC. Then, type I and type II interferon activity scores were calculated for each NPC tumor sample in this study. As shown in **Figure S8A**, interferon activity was suppressed in the RM group (33). Therefore, we guess the 4-gene signature affected prognosis of patients with NPC by increasing cell proliferation and host immune response especially interferon activity.

With the rapid progression of immunological research, the conventional understanding of cancer has been recently refreshed; new immune-related prognostic markers have been identified, and novel therapeutic targets have been developed. Recently, immune subtype-specific signatures were reported by Chen et al. using single-cell transcriptome data, and signatures of some immune cells associated with NPC patient outcomes were also identified (34). However, an understanding of the relationship between the tumor immune landscape and clinical outcome in NPC is still lacking, so we focused on TIICs in NPC. In our study, high levels of immune infiltration were associated with improved clinical outcomes in NPC. In other words, immune escape of tumor cells and immune surveillance of the human immune system have important effects on the prognosis of NPC. PD-1 and PD-L1 serve as immune checkpoints in the tumor microenvironment. Previous studies have reported that PD-1 and PD-L1 are associated with prognosis in some solid tumors (35, 36). Whether the expression of PD-1 and PD-L1 plays a significant role in the prognosis of NPC is still controversial. We compared immune checkpoint gene expression and found that patients with low expression of PD-1 had poor prognosis (**Figures S8B, C**), which was consistent with the study by Cao et al. (37). Moreover, the expression of GLB1L was positively correlated with immune infiltrating level of plasmacytoid dendritic cells, immature dendritic cells, MDSCs, natural killer (NK) cells and central memory CD4^+^ T cells. Among these, immature dendritic cells and central memory CD4^+^ T cells infiltration are significantly associated with NPC prognosis. Notably, some of these infiltrating immune cells, such as CD4^+^ T cells, represent an immune activation. Toor et al. reported that tumor-infiltrating CD4^+^ T cells upregulated PD-1, which is consistent with our study that high expression of PD-1 is associated with good prognosis (38). These findings suggested that GLB1L potentially play a vital role in governing immune cell recruitment to NPC tumors, and thus represent a valuable prognostic biomarker in NPC patients.

One limitation of our study is that the size of the cohort for this study is relatively small. There are few transcriptome data on NPC with prognostic information, so the predictive value of the 4-mRNA signature for prognosis still needs to be validated. Another is that the function of theses gene has not been clarified especially the association with immune cells, so its contribution to the pathogenesis of NPC needs to be revealed in future studies. Despite these limitations, our findings identified that the 4-gene signature be able to predict the outcome of NPC patients and help improve our understanding of TIICs in NPC. We identified distinct biological process underlying the different risk groups; that is, the immune response was differentially regulated in each group, resulting in differential NPC outcomes. Most importantly, our classification scheme can be applied to choose distinct clinical treatment options for NPC patients. For example, patients in the high-risk group may be likely to receive immunostimulants and adjuvants such as interferon. In addition, therapies targeting dysregulated cell cycle progression may also be a good method for these patients. In conclusion, our study identified 4 genes that might be associated with prognosis of NPC and provide a powerful means for predicting NPC patient outcomes. Our analysis also suggests that these genes have a potential role in inducing antitumor immune responses in NPC.

## Materials and Methods

### Data Sources and Study Design

In this study, 3 datasets were analyzed. One dataset was generated by our group in a parallel study (Zhao S. et al, manuscript in submission). The raw sequence data have been deposited in Genome Sequence Archive (https://bigd.big.ac.cn/gsa) and the accession number is HRA000790. This data includes 67 patients with NPC collected from 2013 to 2016 at the Cancer Hospital Chinese Academy of Medical Sciences. 60 NPC samples in total were ultimately included for this study because 6 patients lacked complete follow-up information. Total RNA was isolated from tumor tissues and paired normal tissues using Trizol (Invitrogen) according to manual instruction. RNase H method (Illumina, USA) was used to remove the rRNA. RNA-Sequencing was performed using the HiSeq platform (Illumina). The other two datasets, TCGA-HNSC and GSE102349, comprised 366 HNSC samples and 113 NPC samples, respectively. The TCGA-HNSC dataset was downloaded with the “TCGABiolink” R package. The GSE102349 dataset, which was previously described by Zhang and colleagues (28), was downloaded with the “GEOquery” R package.

### Machine Learning to Identify Significant Genes

A GLM was applied to identify significant genes between the RM and no-RM group. A total of 601 mRNAs were screened after correcting for the effect of age (>= 45 vs < 45) and sex. Two machine learning methods (RF and XGBoost) were carried out by the “RandomForest” and “XGBoost” packages (39). In the RF algorithm, the top 100 mRNAs were selected according to the importance score, and then the intersection of these mRNAs with the mRNAs identified by the XGBoost algorithm was taken. Thirteen mRNAs mostly related to the prognostic classification were eventually selected from the 601 mRNAs.

### Survival Analysis for the Significant Genes

PFS was calculated as the duration from the beginning of treatment to the first relapse or death, and OS was calculated as the duration from the beginning of treatment to death from any cause. The “survminer” and “survival” R packages were used to evaluate the relationship of the expression levels of the 13 significant genes with prognosis (PFS and OS) according to optimal cutoff expression values of the genes.

### Construction of the Prognostic Signature

Univariate Cox analysis was performed to assess the association between the expression of each of the 13 significant genes and OS. Then, predictive markers for survival were identified using a stepwise Cox proportional hazards regression model. A risk score formula was constructed as follows: gene 1 ∗ β1 + gene 2 ∗ β 2 + gene 3 ∗ β3 +···gene n ∗ βn. Gene represents the gene expression level, and β represents the regression coefficient; as such, the risk score was calculated as follows: 0.578 × U2AF1L5 + 2.025 × TMEM265 – 1.19 × GLB1L –1.108 × MLF1. According to the median risk score, patients were classified into a high-risk and a low-risk group. In this analysis, R packages “survminer” and “survival” were used.

### Identification of the Expression Levels of 4-Gene Signature by qRT-PCR

To validate the association of signature and outcome of patients with NPC, we explored the expression level of signature (U2AF1L5, TMEM265, GLB1L and MLF1) using qRT-PCR in ABI7500. Samples were collected from the Cancer Hospital Chinese Academy of Medical Sciences and RNA was isolated using Trizol. Reverse transcription of total RNA was performed with PrimeScript™ RT reagent Kit and TaqMan^®^ Gene Expression Assays (Takara). Gene expression was determined using TaqMan^®^ Fast Advanced Master Mix (Thermo Fisher). All assays were quantified relative to GAPDH.

### ROC Curve and Multivariate Cox Regression Analyses

The prognostic performance was measured using ROC curves. In this analysis, we performed R packages “survivalROC” and “pROC” to plot the curves, calculate the AUC values and 95% confidence interval. The risk score was an independent prognostic factor according to the multivariate Cox regression analysis, with HRs and p values obtained after correcting for age (>= 45 vs < 45), sex, T stage, N stage, and risk score categories.

### Biological Function of 4-Gene Signature

We utilized the R package “clusterProfiler” to determine the biological relevance of the 4-mRNA signature. The gene set “h.all.v7.1.symbols.gmt” was selected and downloaded from the Molecular Signature Database (MSigDB). Gene sets with a p value < 0.05 were considered to be significantly enriched (40).

### The Analysis of Tumor Infiltration Immune Cell

First, we used ESTIMATE algorithm based on the expression level of our NPC RNA-Seq cohort to count the tumor purity, ESTIMATE score, immune score and stromal score of 60 NPC samples using R package “ESTIMATE”. Next, to investigate the difference of immune cell subtypes, ssGSEA was performed to estimate the abundances of 28 immune cell subsets according to the gene expression profiles in the NPC samples using R package “GSVA”. The ssGSEA scores of each immune cells in NPC samples were counted and subsequently were compared between two groups (RM group and no-RM group) with the Wilcoxon test using R package “ggpubr”. The 28 immune cell markers were downloaded from: (https://www.cell.com/cms/10.1016/j.celrep.2016.12.019/attachment/f353dac9-4bf5-4a52-bb9a-775e74d5e968/mmc3.xlsx) (41). Besides, we used correlation analysis to evaluate the correlation between the expression levels of 4-gene signature and ssGSEA scores of each tumor infiltrating immune cells in 60 NPC samples.

### Type I and Type II Interferon Activity Scores

To determine the type I and type II interferon activity scores for the 60 NPC tumor samples, we calculated the sum of the z-scores for the genes in the type I and type II interferon pathways as previously described (33).

### Statistical Analysis

The statistical analyses were mainly completed using R version 3.5.2 (http://www.r-project.org). The Wilcoxon test was performed to compare the expression levels of genes between different groups. Survival curves were generated by the K-M method, and p values were calculated based on the log-rank test. Pearson correlation analysis was performed to evaluate the correlation between the expression levels of two genes or the expression levels of genes and TIICs. Other bioinformatics analyses were performed with several R packages. A p value less than 0.05 was considered to indicate significance in this study.

## Data Availability Statement

RNA-Seq data has been deposited in publicly accessible repositories. The data can be found here: https://ngdc.cncb.ac.cn/search/?dbId=&q=PRJCA004867. The assigned accession of the submission is: HRA000790.

## Ethics Statement

Written informed consent was obtained from the individual(s), and minor(s)’ legal guardian/next of kin, for the publication of any potentially identifiable images or data included in this article.

## Author Contributions

SZ designed the study, analyzed the RNA-Seq data, and drafted the manuscript. XD completed the data acquisition from NPC RNA-Seq, analysis and interpretation of data, added the follow-up verification of another cohort and validated the model. XGN completed the endoscopy, collected the NPC specimens and helped with acquisition of clinical data. LL double-checked pathological diagnosis and helped with analyzing the data. XL contributed to the conception for the work and provided scientific guidance during part of the study. KTZ and YNG conceived the study, revised the manuscript and performed project administration. All authors contributed to the article and approved the submitted version.

## Conflict of Interest

The authors declare that the research was conducted in the absence of any commercial or financial relationships that could be construed as a potential conflict of interest.

## Publisher’s Note

All claims expressed in this article are solely those of the authors and do not necessarily represent those of their affiliated organizations, or those of the publisher, the editors and the reviewers. Any product that may be evaluated in this article, or claim that may be made by its manufacturer, is not guaranteed or endorsed by the publisher.
