# Supplementary material for: Exploration of a Novel Prognostic Risk Signature and Its Effect on the Immune Response in Nasopharyngeal Carcinoma
Source: Front Oncol. 2021 Oct 7;11:709931. doi: 10.3389/fonc.2021.709931 (PMC8529178; doi:10.3389/fonc.2021.709931)
Supplement: Supplementary file 1 [file DataSheet_1.docx]

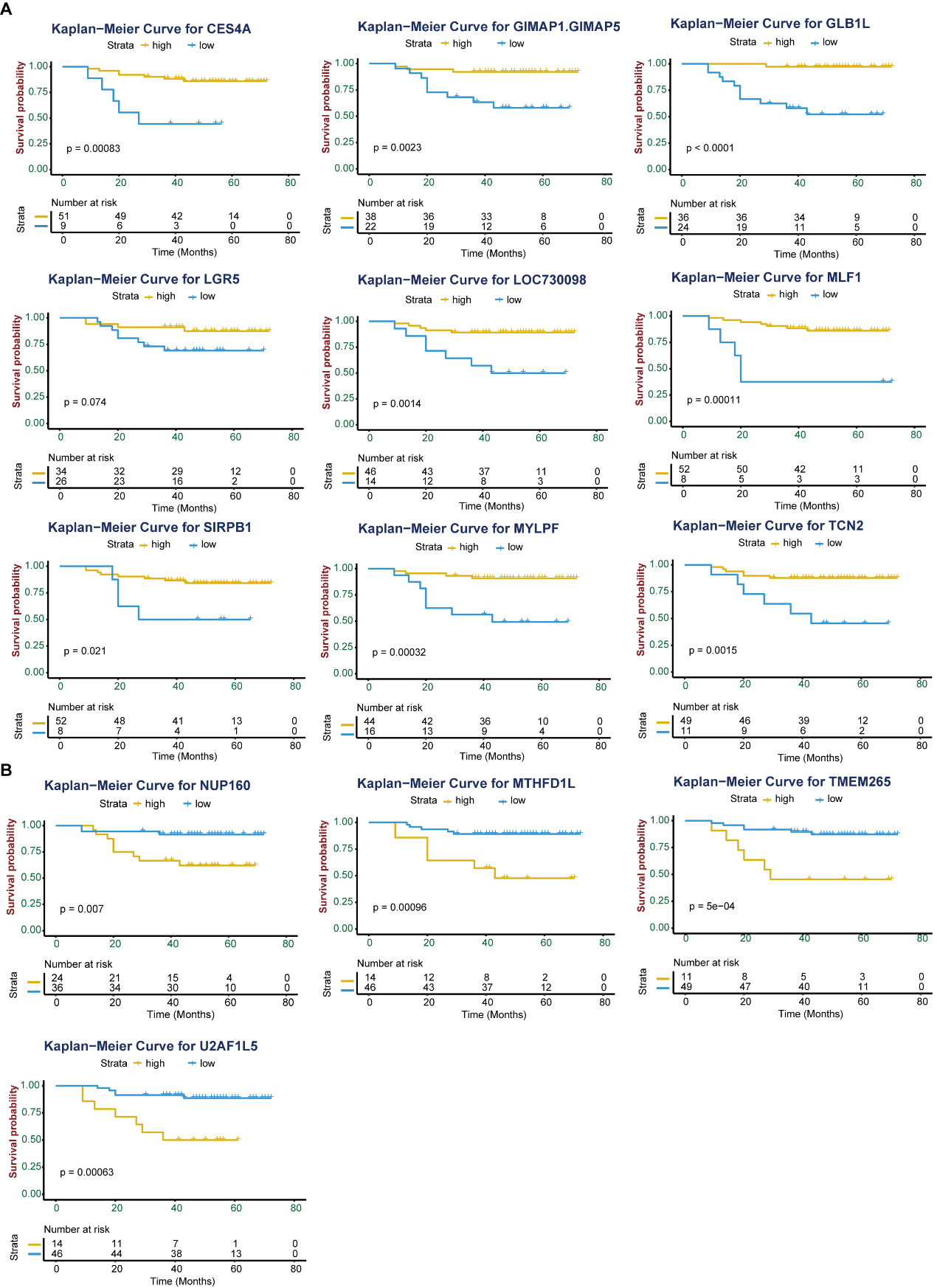


**Figure S1.** Kaplan-Meier curves for overall survival (OS) for high and low expression of the 13 genes in NPC patients. **(A)** Kaplan-Meier survival curves showing that high expression of the genes CES4A, GIMAP1-GIMAP5, GLB1L, LGR5, LOC730098, MLF1, SIRPB1, MYLPF, and TCN2 was associated with improved OS in patients with NPC. **(B)** Kaplan-Meier survival curves showing that high expression of the genes NUP160, MTHFD1L, TMEM265, and U2AF1L5 was associated with poor OS in patients with NPC. The Kaplan-Meier p values are shown.


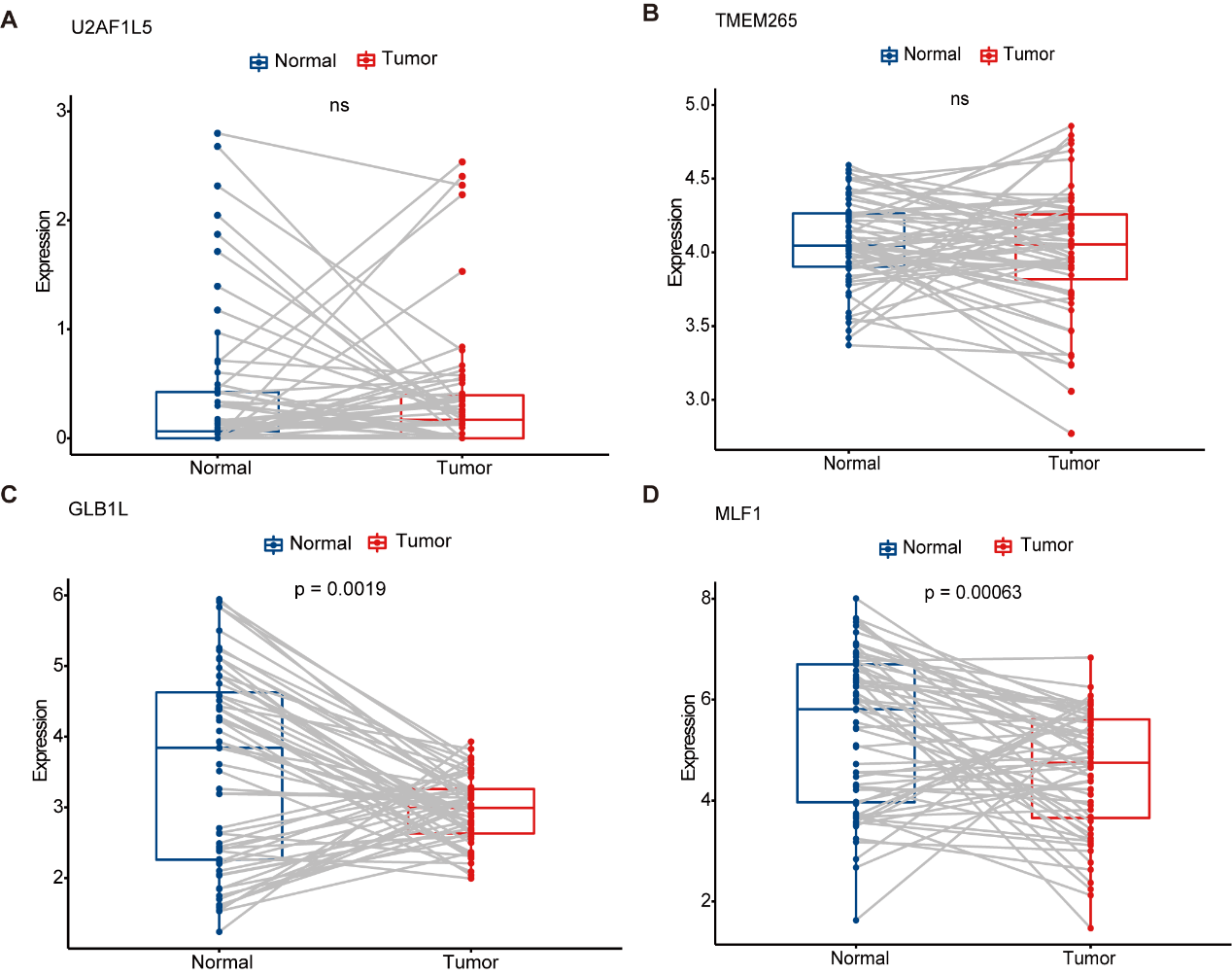


**Figure S2.** Expression of the 4 mRNAs in the signature between paired NPC tumor and normal tissues. **(A)** The expression of U2AF1L5 was not different between tumor and normal tissues. **(B)** The expression of TMEM265 was not different between tumor and normal tissues. **(C)** The expression of GLB1L was different between tumor and normal tissues (p = 0.0019). **(D)** The expression of MLF1 was different between tumor and normal tissues (p = 0.00063).


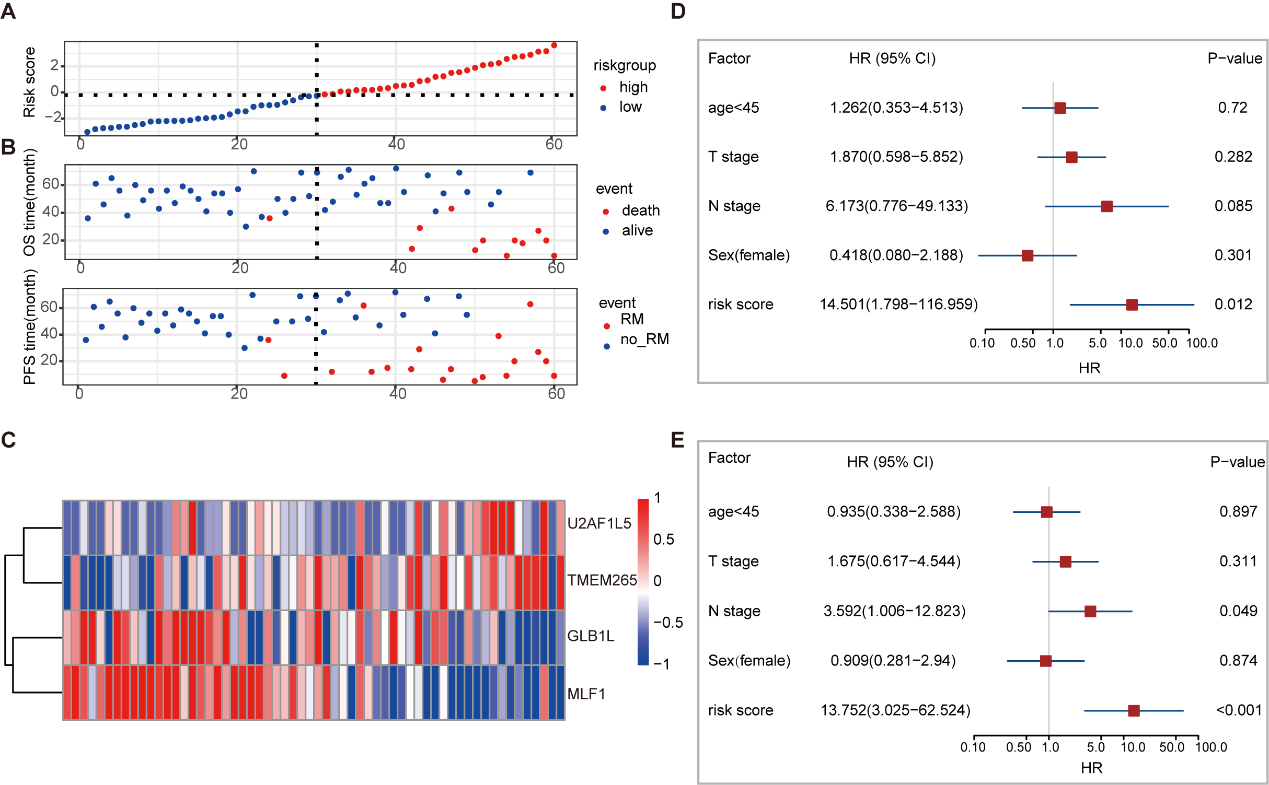


**Figure S3.** Correlation between the 4-mRNA signature risk score and the prognosis of patients with NPC. **(A)** Distribution of the risk scores of NPC patients. **(B)** Top: red represents nonsurvivors, blue represents survivors. Bottom: red represents the RM group, and blue represents the no-RM group. The dotted line represents the median value of the risk score. **(C)** Heatmap of the 4-mRNA signature in 60 patients with NPC. **(D)** Forest plots of the multivariate Cox regression analysis results for OS, including the risk score and some clinical variables (risk score: HR = 14.501, p = 0.012). **(E)** Forest plots of the multivariate Cox regression analysis results for PFS (risk score: HR = 13.752, p < 0.001; age: < 45 vs. ≥45 years; sex: female vs. male; T stage: T4 vs. T1-3; N stage: N2-3 vs. N0-1; risk score: high risk vs. low risk).


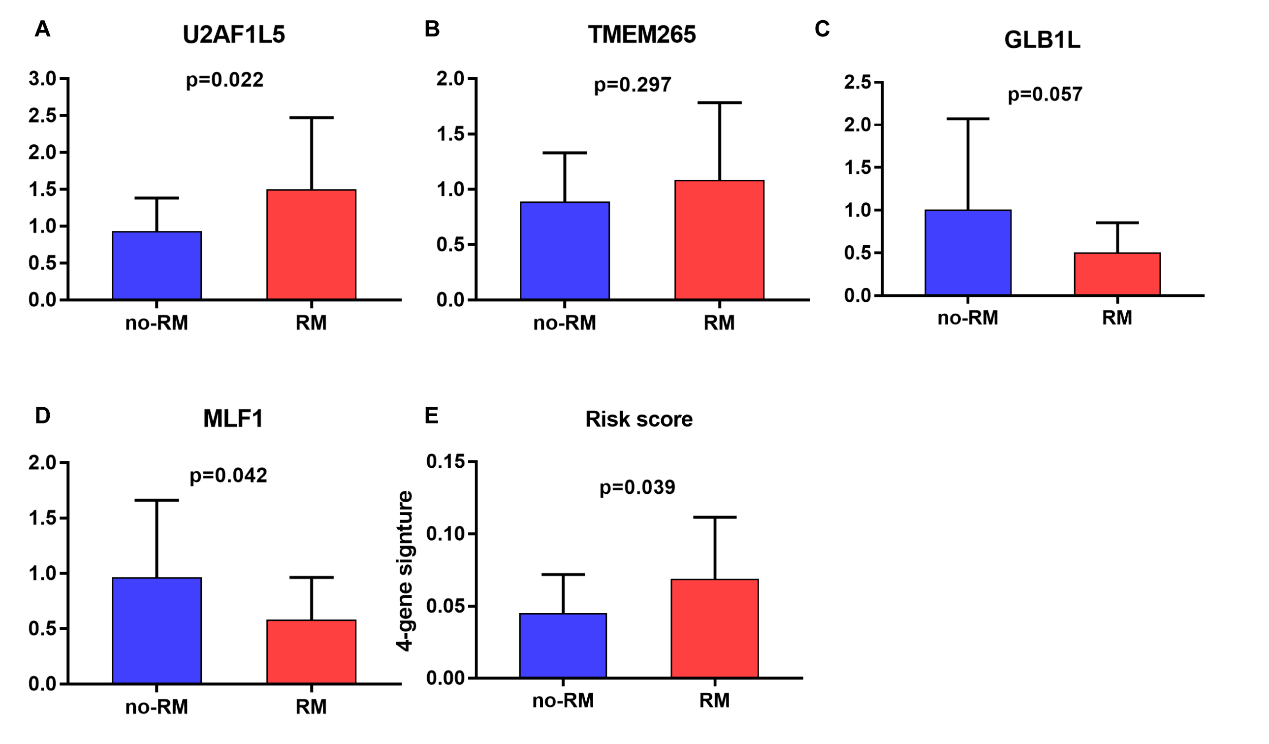


**Figure S4.** Expression levels of 4-gene signature between no-RM and RM group in the qRT-PCR cohort of NPC. (A) Expression level of U2AF1L5. (B) Expression level of TMEM265. (C) Expression level of GLB1L. (D) Expression level of MLF1. (E) The risk score in the qRT-PCR cohort based on 4-gene signature prognostic model.


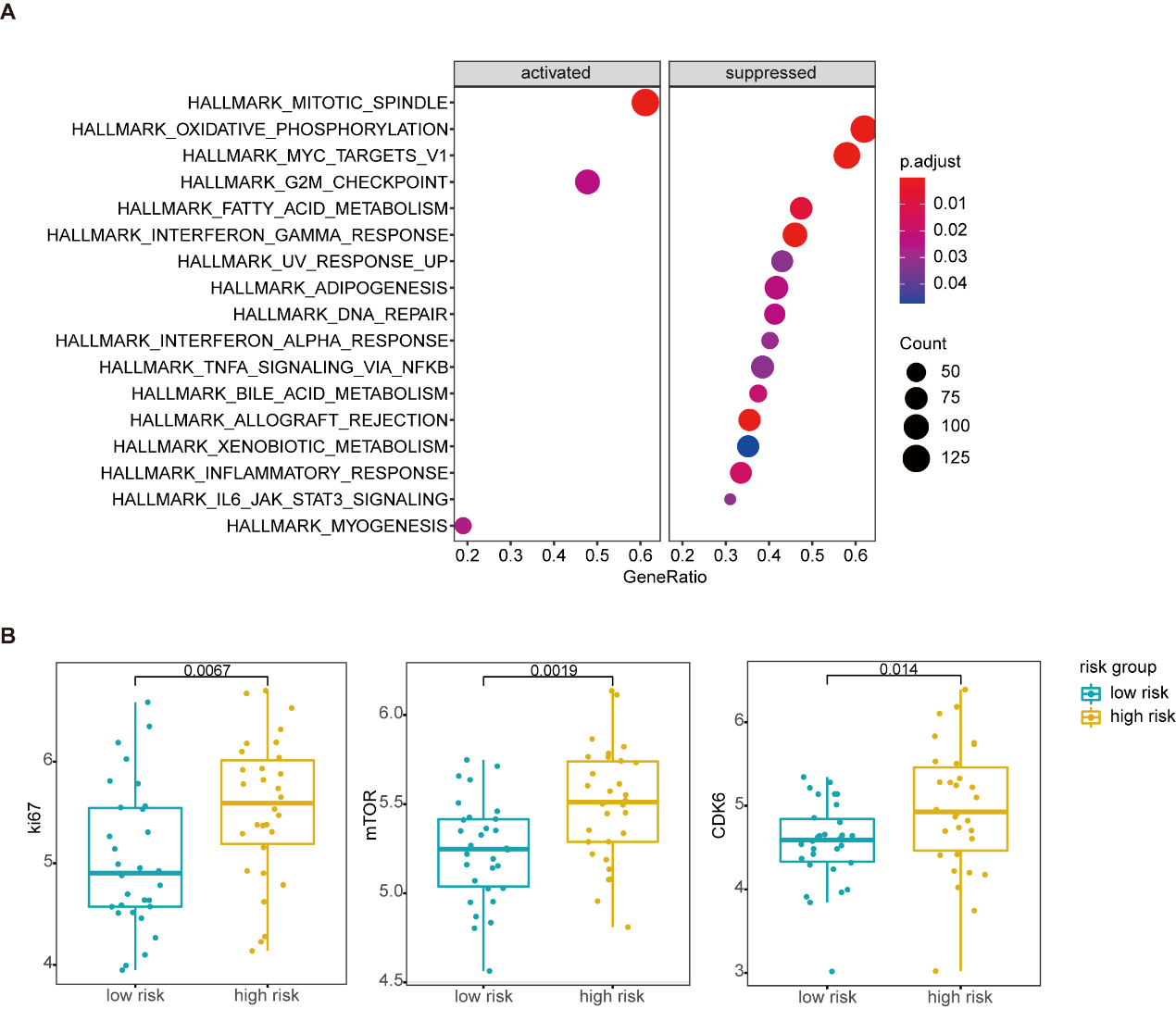


**Figure S5.** Bioinformatics analysis of the biological relevance of the signature among patients in different risk groups. **(A)** Hallmark gene set enrichment analysis comparing the high- and low-risk groups. **(B)** Cell proliferation-related gene (Ki67) and cell cycle-related gene (mTOR and CDK6) expression levels between the low-risk and high-risk groups.


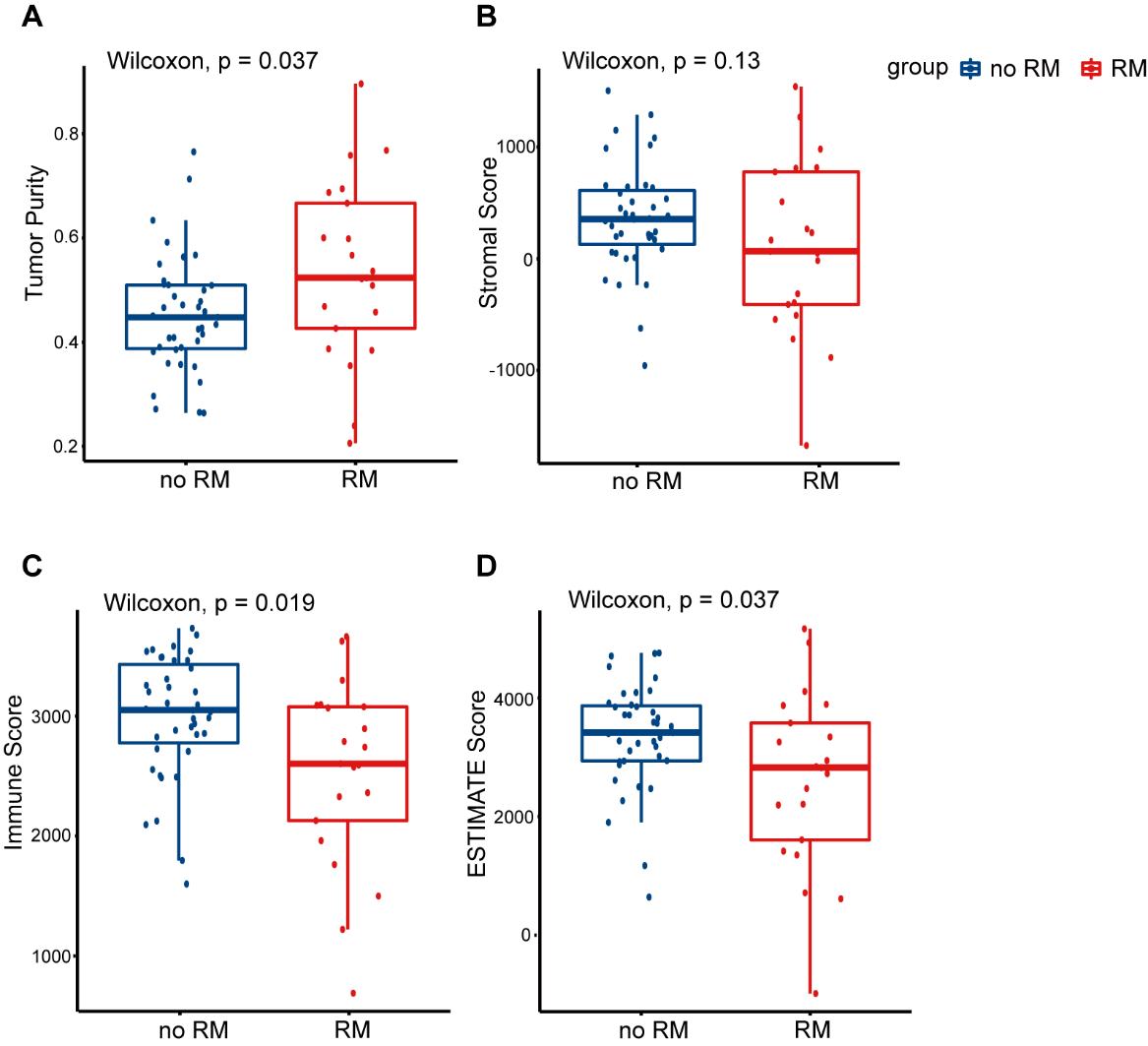


**Figure S6.** (A) Tumor purity, (B) stromal scores, (C) immune scores and (D) ESTIMATE scores in the no-RM and RM groups.


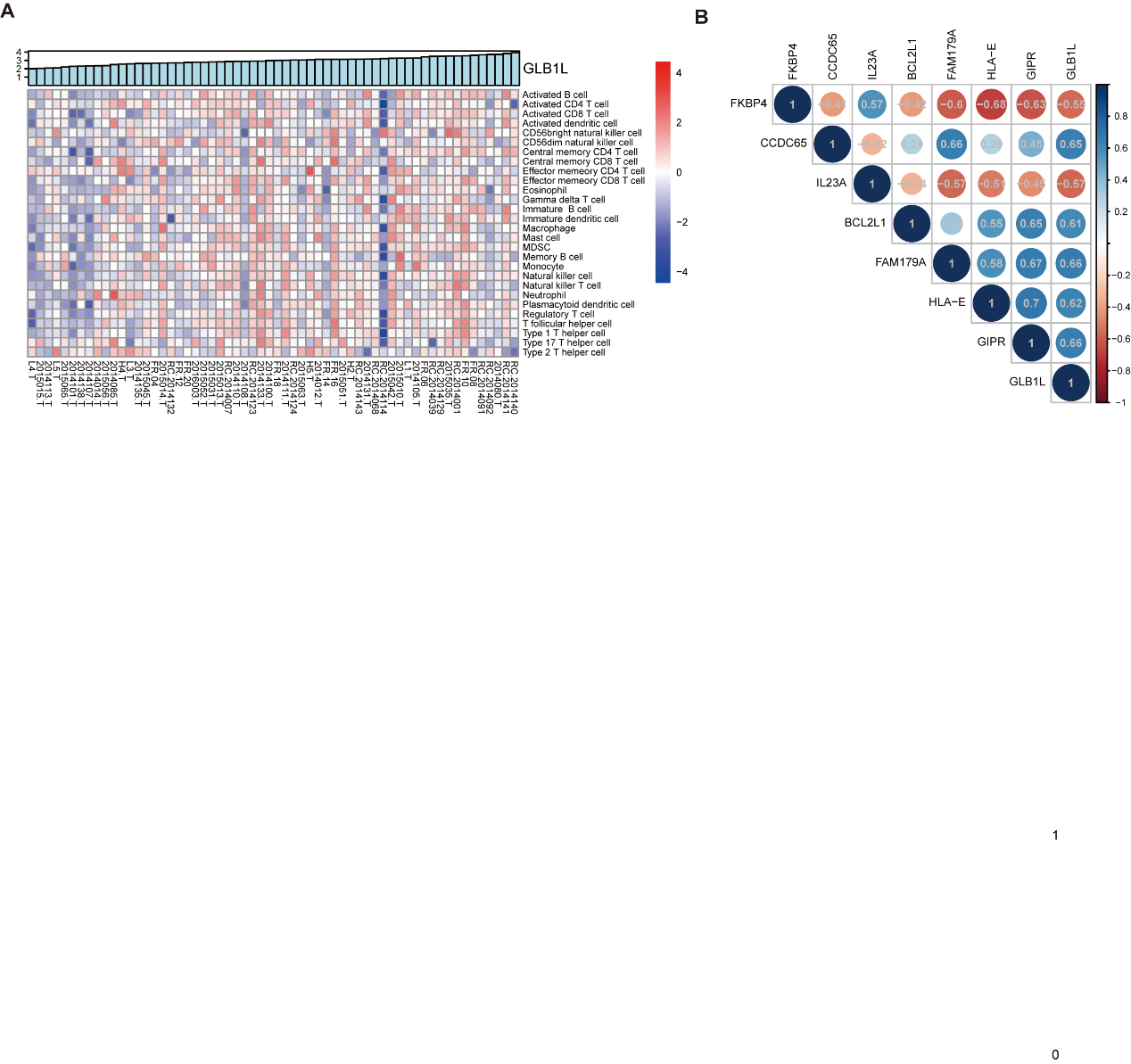


**Figure S7.** Association of GLB1L expression with infiltrating immune cells. **(A)**. The NESs of all immune infiltration components and the expression level of GLB1L in NPC. **(B)** Correlation analysis of GLB1L expression and immune cell markers.


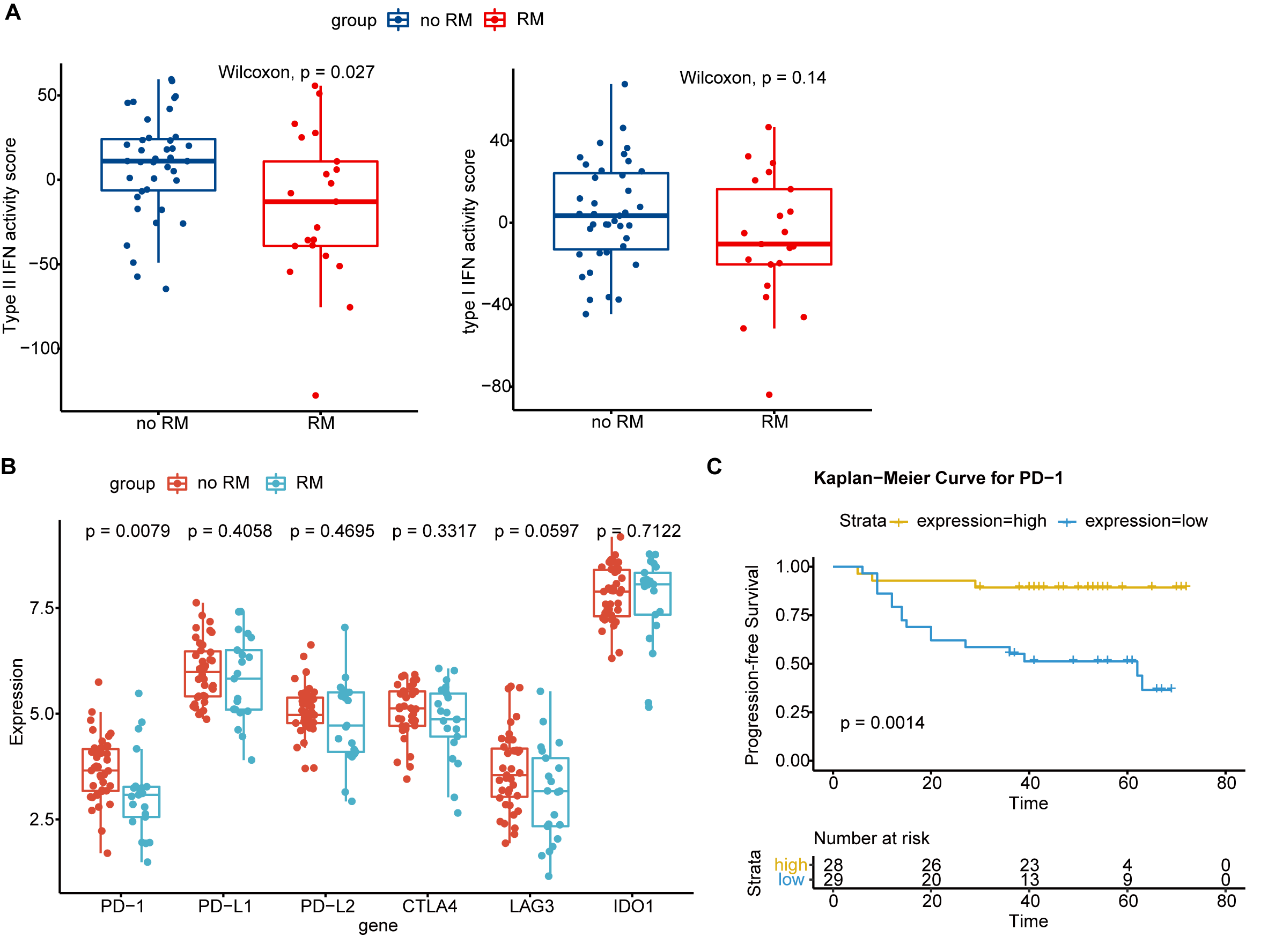


**Figure S8.** Association between the immune response (IFN activity and immune checkpoint genes) and prognosis in NPC. (**A**) Type I (IFN-α and IFN-β) and type II (IFN-γ) interferon activity scores for the no-RM and RM groups. **(B)** Evaluation of the expression of immune checkpoint genes (PD-1, PD-L1, PD-L2, CTLA4, LAG3 and IDO1) in the no-RM and RM groups. **(C)** Kaplan-Meier survival plots showing the association between the expression level of PD-1 and PFS in patients with NPC. The Kaplan-Meier p value was 0.0014.
